# Supplementary material for: Interventions to Reduce Implicit Bias in High-Stakes Professional Judgements: A Systematic Review
Source: Behav Sci (Basel). 2025 Nov 20;15(11):1592. doi: 10.3390/bs15111592 (PMC12649508; doi:10.3390/bs15111592)
Supplement: Supplementary file 1 [file behavsci-15-01592-s001.zip › behavsci-3893774-supplementary.pdf]

Supplementary Material (S1) – Search Strategy (March 2025)

This file accompanies Section 2.1 (Search Strategy) and provides the full database-specific search configurations corresponding to the general strategy described in the manuscript.

This document provides a complete, database-specific record of the systematic search conducted in March 2025 through Goldsmiths, University of London's institutional access. All Boolean strings, field scopes, and filters correspond to the exact configurations allowed within each database interface. Filters were applied using the platforms' built-in limiter menus and were constrained to the available options within each database (e.g., 'Peer-reviewed' where available, 'Adults' and 'Humans' only where databases supported demographic filtering). No custom or external filters were applied.

1. APA PsycINFO

Platform / Interface: EBSCOhost (research.ebsco.com).

Field searched: All fields (TX – All Text), including Title, Abstract, Author Keywords, and Subject Headings.

Search string:

("implicit" OR "unconscious" OR "subconscious" OR "automatic" OR "heuristic" OR "myth*") AND ("bias*" OR "prejudic*" OR "stereotyp*" OR "attitude*" OR "association*" OR "discriminat*" OR "preference*" OR "myth*") AND ("debias*" OR "intervention*" OR "reduc*" OR "training" OR "strategy" OR "method" OR "program*" OR "approach*" OR "chang*" OR "tool*" OR "educat*" OR "modif*" OR "diminish*" OR "counteract*" OR "mitigat*" OR "refram*" OR "effective*") AND ("decision-making" OR "decision task" OR "judg*" OR "evaluat*" OR "deliberation*" OR "verdict")

Filters applied (database-supported): Peer-reviewed; Journal Article; English language; Year range 2000–2025; Population: Adults (18+ years & older, all categories available), Humans. Excluded: Dissertations. These options were directly selected from EBSCOhost limiters.

Results retrieved: 1,647.

2. APA PsycArticles

Platform / Interface: EBSCOhost (research.ebsco.com).

Field searched: All fields (TX – All Text), including Title, Abstract, Author Keywords, and Subject Headings.

Search string: Identical to APA PsycINFO, entered as four concept blocks combined using AND.

Filters applied (database-supported): Peer-reviewed; Journal Article; English; 2000–2025; Adults (18+ years & older, all categories available); Humans. All limiters were applied through EBSCOhost menus.

Results retrieved: 167.

3. Criminology Collection

Platform / Interface: ProQuest (proquest.com).

Field searched: Anywhere except full text (NOFT); the broadest metadata field in ProQuest, encompassing title, abstract, subject terms, and author keywords, but excluding full text.

Search structure:

("implicit" OR "unconscious" OR "subconscious" OR "automatic" OR "heuristic" OR "myth*") AND ("bias*" OR "prejudic*" OR "stereotyp*" OR "attitude*" OR "association*" OR "discriminat*" OR "preference*" OR "myth*") AND ("debias*" OR "intervention*" OR "reduc*" OR "training" OR "strategy" OR "method" OR "program*" OR "approach*" OR "chang*" OR "tool*" OR "educat*" OR "modif*" OR "diminish*" OR "counteract*" OR "mitigat*" OR "refram*" OR "effective*") AND ("decision-making" OR "decision task" OR "judg*" OR "evaluat*" OR "deliberation*" OR "verdict")

Filters applied (database-supported): Peer-reviewed; Journal Article; English; Publication date after 31 Dec 1999; Duplicate removal via ProQuest's 'Exclude duplicate documents' option. No manual filters applied.

Results retrieved: 179.

4. ERIC

Platform / Interface: ProQuest (proquest.com).

Field searched: Anywhere except full text (NOFT), encompassing title, abstract, subject terms, and author keywords.

Search string: Same Boolean string as Criminology Collection.

Filters applied (database-supported): Peer-reviewed; Journal Article; English; Publication date after 31 Dec 1999. No manual filters applied.

Results retrieved: 365.

5. Social Science Database

Platform / Interface: ProQuest (proquest.com).

Field searched: Anywhere except full text (NOFT)"; the broadest metadata field in ProQuest, encompassing title, abstract, subject terms, and author keywords, but excluding full text.

Search string: Same Boolean string as Criminology Collection.

Filters applied (database-supported): Peer-Reviewed; Source type = Scholarly Journals; Language = English; Publication years 2000–2029. No manual filters applied.

Results retrieved: 474.

6. ASSIA (Applied Social Sciences Index and Abstracts)

Platform / Interface: ProQuest (proquest.com).

Field searched: Anywhere except full text (NOFT)"; the broadest metadata field in ProQuest, encompassing title, abstract, subject terms, and author keywords, but excluding full text.

Search string: Same Boolean string as Criminology Collection.

Filters applied (database-supported): Peer-Reviewed; Scholarly Journals; English; Publication years 2000–2029. No manual filters applied.

Results retrieved: 561.

7. PubMed

Platform / Interface: PubMed Advanced Search Builder (pubmed.ncbi.nlm.nih.gov).

Field searched: Title/Abstract for all search terms (as selected in the dropdown).

Search structure:

("implicit"[Title/Abstract] OR "unconscious"[Title/Abstract] OR "subconscious"[Title/Abstract] OR "automatic"[Title/Abstract] OR "heuristic"[Title/Abstract] OR "myth*"[Title/Abstract]) AND ("bias*"[Title/Abstract] OR "prejudic*"[Title/Abstract] OR "stereotyp*"[Title/Abstract] OR "attitude*"[Title/Abstract] OR "association*"[Title/Abstract] OR "discriminat*"[Title/Abstract] OR "preference*"[Title/Abstract] OR "myth*"[Title/Abstract]) AND ("debias*"[Title/Abstract] OR "intervention*"[Title/Abstract] OR "reduc*"[Title/Abstract] OR "training"[Title/Abstract] OR "strategy"[Title/Abstract] OR "method"[Title/Abstract] OR "program*"[Title/Abstract] OR "approach*"[Title/Abstract] OR "chang*"[Title/Abstract] OR "tool*"[Title/Abstract] OR "educat*"[Title/Abstract] OR "modif*"[Title/Abstract] OR "diminish*"[Title/Abstract] OR "counteract*"[Title/Abstract] OR "mitigat*"[Title/Abstract] OR "refram*"[Title/Abstract] OR "effective*"[Title/Abstract]) AND ("decision-making"[Title/Abstract] OR "decision task"[Title/Abstract] OR "judg*"[Title/Abstract] OR "evaluat*"[Title/Abstract] OR "deliberation*"[Title/Abstract] OR "verdict"[Title/Abstract])

Filters applied (database-supported): Language = English; Species = Humans; Age = Adult (18+ years, all categories available); Publication dates 2000–2025; Excluded preprints using PubMed's publication type filter.

Results retrieved: 1,787.

8. Web of Science

Platform / Interface: Clarivate Web of Science Advanced Search.

Field searched: Topic (TS) – searches Title, Abstract, Author Keywords, and Keywords Plus.

Search structure:

TS=("implicit" OR "unconscious" OR "subconscious" OR "automatic" OR "heuristic" OR "myth*") AND

TS=("bias*" OR "prejudic*" OR "stereotyp*" OR "attitude*" OR "association*" OR "discriminat*" OR "preference*" OR "myth*") AND

TS=("debias*" OR "intervention*" OR "reduc*" OR "training" OR "strategy" OR "method" OR "program*" OR "approach*" OR "chang*" OR "tool*" OR "educat*" OR "modif*" OR "diminish*" OR "counteract*" OR "mitigat*" OR "refram*" OR "effective*") AND

TS=("decision-making" OR "decision task" OR "judg*" OR "evaluat*" OR "deliberation*" OR "verdict")

Filters applied (database-supported): Article; English; 1999-12-31 – 2026-01-01. No manual filters applied.

Results retrieved: 12,557

***Notes:**

• Each database was searched through its institutional interface (EBSCOhost, ProQuest, PubMed, or Clarivate Web of Science).

• The search strings above reproduce the syntax entered into the search boxes.

• Field scopes correspond roughly to Title + Abstract + Subject headings + Keyword as supported by available database-limiters within their Advanced Search filter panels.

• Filters were applied only to the extent permitted by the database's limiter menus. No custom filters or cross-database exclusions were imposed manually.

• All searches were completed in March 2025.
